# The Impact of Histology Subtype and Size of Giant Retroperitoneal Liposarcomas on Their Risk of Recurrence: A Retrospective Cohort Analysis

**DOI:** 10.3390/cancers18101649

**Published:** 2026-05-20

**Authors:** Domenico Santangelo, Agostino Fernicola, Armando Calogero, Martina Sommese, Antonio Miele, Luca Carlomagno, Andrea Paolillo, Alessio Cece, Domenica Pignatelli, Antonio Alvigi, Luigi Ricciardelli, Alberto Servetto, Massimo Imbriaco, Nicola Carlomagno, Michele Santangelo, Alfonso Santangelo

**Affiliations:** 1 Department of Advanced Biomedical Sciences, University of Naples Federico II, 80131 Naples, Italy; santangelo.domenico@hsr.it; 2 Department of Radiology, IRCCS San Raffaele Hospital, Via Olgettina 60, 20132 Milan, Italy; 3 Operative Unit of General and Emergency Surgery, Department of Advanced Biomedical Sciences, University of Naples Federico II, 80131 Naples, Italy; agostinofernicola@yahoo.it (A.F.); armando.calogero2@unina.it (A.C.); sommesemartina@gmail.com (M.S.); antonio.miele97@gmail.com (A.M.); luca.carlomagno0410@gmail.com (L.C.);andrea.paolillo@unina.it (A.P.); nicola.carlomagno@unina.it (N.C.); michele.santangelo@unina.it (M.S.); 4 Department of Integrated Activities in Surgery, Orthopedy and Hepato-Gastroenterology, University of Campania “Luigi Vanvitelli”, 80138 Naples, Italy; alessio.cece@unicampania.it; 5 Department of Clinical Medicine and Surgery, University of Naples Federico II, 80131 Naples, Italy; domenica.pignatelli@unina.it (D.P.); antonio.alvigi@unina.it (A.A.); 6 Unit of Emergency Surgery, Dei Colli Hospital, CTO Hospital, 80131 Naples, Italy; ricciardelliluigi@gmail.com; 7 Operative Unit of Oncology, Department of Clinical Medicine and Surgery, University of Naples Federico II, 80131 Naples, Italy; alberto.servetto@unina.it; 8 Operative Unit of Diagnostic Imaging and Radiotherapy, Department of Advanced Biomedical Sciences, University of Naples Federico II, 80131 Naples, Italy; massimo.imbriaco@unina.it; 9 Division of Oncology, Unit of Urology, IRCCS Ospedale San Raffaele, Vita-Salute San Raffaele University, 20132 Milan, Italy

**Keywords:** retroperitoneal sarcoma, liposarcoma, giant tumor, histology, recurrence, prognosis

## Abstract

**Introduction**: Giant retroperitoneal liposarcomas (GRPLs) are rare tumors that often reach considerable size before diagnosis due to their indolent growth and deep anatomical location. Surgery represents the only potentially curative treatment, yet recurrence rates remain high. While histological subtype is a recognized predictor of recurrence, the prognostic role of tumor size, particularly in giant tumors, remains controversial. This study evaluates the impact of tumor size and histological subtype on recurrence risk in a literature-based retrospective cohort. **Materials and Methods**: Data were extracted from a literature-based database of GRLPs published between 2004 and 2023. Only tumors >20 cm treated without positive surgical margins were included; patients receiving adjuvant therapy or with missing follow-up were excluded. Histological subtype (well-differentiated vs. other) was the main variable of interest. Recurrence-free survival (RFS) was defined as the primary endpoint and estimated using the Kaplan–Meier method. The association between histological subtype and recurrence risk was evaluated using a Cox proportional hazards model. A sensitivity analysis was performed to explore the potential interaction between tumor size and histological subtype. **Results**: Our final cohort yielded a total of 81 patients, of whom 47 (58%) had a well-differentiated GRLPs. The median tumor size was 38 cm and median follow-up was 16 months, with 24 recurrences observed. At 24 months, RFS was higher in well-differentiated tumors than in other histological subtypes (81% vs. 41%). In multivariable Cox analysis, histology was independently associated with recurrence risk (HR 3.2, 95% CI 1.28–8.17, *p* = 0.01), whereas tumor size showed no association with recurrence. Interaction analysis confirmed no differential effect of tumor size across histological subtypes. **Conclusions**: In this literature-based cohort of GRLPs treated with surgery, histological subtype independently predicted recurrence, whereas tumor size showed no prognostic value, either overall or within individual histological subtypes.

## Simple Summary

Giant retroperitoneal liposarcomas are rare soft-tissue tumors that frequently recur after surgical resection, yet the relative contribution of tumor size and histological subtype to recurrence risk remains unclear. In this retrospective literature-based cohort study, we evaluated whether tumor dimension influences recurrence-free survival in patients with giant retroperitoneal liposarcoma and whether this effect differs according to histology. Our analysis showed that histological subtype, rather than tumor size, was the principal determinant of recurrence. In particular, tumors with non-well-differentiated histology were associated with a substantially higher risk of recurrence than well-differentiated tumors, whereas tumor dimension did not significantly affect recurrence risk overall or within histological subgroups. These findings suggest that, even among very large tumors, biological behavior may be more clinically relevant than size for postoperative risk stratification and patient follow-up planning.

## 1. Introduction

Retroperitoneal liposarcomas (RPLs) are rare mesenchymal malignancies arising from adipocytic tissues within the retroperitoneal space [1,2,3,4]. These tumors have an estimated incidence of approximately 0.5 per 100,000 individuals per year [3], with a peak occurrence in the seventh and eighth decades of life and higher incidence in the male population [5]. According to the 2020 World Health Organization (WHO) [6] classification of soft-tissue tumors, liposarcomas are divided into five main histological subtypes: well-differentiated, dedifferentiated, myxoid, pleomorphic, and myxoid pleomorphic liposarcoma. Although uncommon, they represent one of the most frequent histological subtypes among retroperitoneal sarcomas. Because of their deep anatomical location, slow growth pattern, and often indolent clinical course, these tumors may remain asymptomatic for a prolonged period and can reach a remarkable size before diagnosis. For this reason, many of these lesions are described as “giant” retroperitoneal liposarcomas (GRPLs). Despite the growing use of this definition in the literature, there is still no universally accepted dimensional threshold for classifying a retroperitoneal liposarcoma as giant. Different staging and classification systems have been proposed, including the American Joint Committee on Cancer (AJCC, 9th edition) [7] and the Vanderbilt staging system [8], yet no clear consensus has emerged regarding the most appropriate size cut-off for defining GRPLs. This lack of standardization limits comparisons across studies and contributes to the heterogeneity of available evidence. Complete surgical resection remains the cornerstone of treatment for localized RPLs and is currently regarded as the main potentially curative option. However, surgery for these tumors is often highly demanding, particularly in the setting of very large masses. The retroperitoneum is a complex anatomical compartment containing major vessels, kidneys, ureters, pancreas, and other vital structures, all of which may be displaced, compressed, or directly involved by the tumor. As tumor size increases, the technical complexity of the operation generally rises accordingly, often requiring multivisceral resection and extensive surgical planning [9,10]. Even so, complete macroscopic excision with negative margins is frequently pursued, including in patients with giant tumors, whenever technically feasible and oncologically appropriate. Despite aggressive surgical management and advances in perioperative care, recurrence remains a major clinical problem in patients with RPLs. Reported recurrence rates remain high, reaching up to 60% in some series [11], with local recurrence in particular representing the predominant pattern of treatment failure. Furthermore, recurrence often develops relatively early, with many patients experiencing disease relapse within the first two years after surgery [12]. This high propensity for recurrence has important implications for prognosis, follow-up strategies, and therapeutic decision-making. Identifying the factors associated with recurrence risk is therefore essential to improve postoperative risk stratification and better tailor surveillance protocols. Among the variables that have been investigated, histological subtype is widely recognized as one of the most relevant prognostic factors in RPLs [13,14,15,16]. In particular, well-differentiated liposarcomas generally display a more indolent biological behavior, whereas dedifferentiated and other non-well-differentiated subtypes are associated with more aggressive disease, a higher likelihood of recurrence, and worse oncological outcomes [17,18,19,20]. By contrast, the prognostic role of tumor size remains less clear [13]. Although larger tumors are intuitively assumed to reflect more advanced disease and greater biological aggressiveness, several reports have failed to demonstrate tumor dimension as an independent predictor of recurrence [21,22]. Thus, while size undoubtedly affects surgical complexity, its actual impact on recurrence-free survival remains controversial. This issue is even more uncertain in the specific subgroup of giant retroperitoneal liposarcomas. Once tumors have already exceeded a very large size, it is unclear whether further differences in dimension continue to influence recurrence risk or whether tumor biology becomes the dominant determinant of outcome. Moreover, the potential interaction between tumor size and histological subtype has not been thoroughly explored. It is plausible that size may have a different prognostic significance according to histology, but this hypothesis has not been adequately investigated in previous studies, largely because of the rarity of the disease and the limited size of individual institutional series. To address this gap in knowledge, we conducted a retrospective literature-based cohort study focused on giant retroperitoneal liposarcomas. The aim of the study was to evaluate the respective impact of tumor size, and histological subtype on recurrence risk in this uncommon clinical setting. In particular, we sought to determine whether size retains prognostic significance among already giant tumors and whether its effect differs across histological categories. By analyzing a large cohort derived from the published literature, we aimed to clarify the relative contribution of dimensional and pathological factors to recurrence-free survival and to provide additional evidence that may support prognostic assessment and postoperative management in patients with GRPLs.

## 2. Materials and Methods

### 2.1. Data Sources and Population

We extracted data from an internal database originally developed for a systematic review focused on GRPLs [23]. This database included all published case reports and case series on GRPLs between 2004 and 2023 for which a full-text English version was available. The methodology adopted for the literature search, study selection, and data extraction has been previously described in detail in the original publication, including the corresponding PRISMA flowchart. In line with prior reports and commonly adopted definitions, only tumors measuring more than 20 cm in maximum diameter were considered eligible for inclusion in the present study. Demographic, clinical, pathological, surgical, and follow-up variables were retrieved whenever available from the original manuscripts. To reduce potential confounding factors affecting oncological outcomes, patients who underwent incomplete surgical resection (R1 or R2) or who received adjuvant chemotherapy and/or radiotherapy were excluded from the final analysis. The rationale of excluding patients receiving adjuvant treatment comes from two specific assumptions: First, the inclusion of patients treated with adjuvant therapy would have introduced a substantial treatment-related confounding bias, as the administration of adjuvant treatment is itself a consequence of perceived higher recurrence risk. Second, and most critically, adjuvant therapy directly modifies the natural history of recurrence, making it impossible to disentangle the independent prognostic contribution of histological subtype and tumor size from the treatment effect itself. Finally, patients lacking adequate follow-up information or recurrence data were also excluded to ensure the reliability and consistency of survival analyses. A flowchart explaining the selection criteria and a table comparing included and excluded patients are reported in the Supplementary Materials (Figure S1 and Table S1).

### 2.2. Variables and Endpoint

Our primary variable of interest was histological subtype. In particular, the analysis focused on the comparison between well-differentiated giant retroperitoneal liposarcomas (WD-GRPLs) and tumors with other histological subtypes, including all other histological subtypes defined by the WHO classification [6]. This distinction was selected based on the well-established biological and prognostic differences reported among liposarcoma subtypes in the existing literature. Histological classification was retrieved from the original reports and categorized accordingly for the purpose of the analysis.

To better characterize the study population and account for potential baseline differences within the examined cohort, several demographic and clinicopathological variables were extracted whenever available. These included patient age at surgery, sex, maximum tumor diameter, histological subtype, and recurrence status during follow-up. Tumor size was considered both as a continuous variable and as a clinically relevant characteristic potentially associated with surgical complexity and oncological outcomes.

The primary study endpoint was recurrence-free survival (RFS), defined as the time interval between surgical tumor excision and the diagnosis of either local or distant recurrence, as reported in the original publication. Patients who did not experience recurrence were censored at the time of the last available follow-up. Both local and metastatic recurrences were included in the definition of the endpoint in order to provide a comprehensive assessment of disease recurrence after surgery.

### 2.3. Statistical Analysis

Descriptive statistics consisted of median and interquartile range (IQR) for continuous variables, whereas categorical variables were summarized using frequencies and percentages. Strata were compared using the chi-square test and the Wilcoxon rank sum test for categorical and numerical variables, respectively. Due to the different epidemiological distribution across genders, patients were stratified both by histology and sex. Kaplan–Meier curves were used to depict RFS after stratifying patients by histology (WD-GRPLs vs. other histology). A Cox regression analysis was performed to evaluate the impact of histology on RFS, adjusting for the tumor dimension and sex. An exploratory interaction analysis was subsequently carried out to account for the possible differential effect of size on either WD or other histology GRPLs’ risk of recurrence. All statistical tests were performed using R v.3.0.2 (R Foundation for Statistical Computing, Vienna, Austria; www.r-project.org). An alpha of 0.05 was used, with any *p*-value < alpha considered significant.

## 3. Results

### 3.1. Demographic Information and Clinical Variables

Our cohort yielded a total of 81 patients, 47 (58%) of whom were diagnosed with a WD-GRPL. Median age was 58 years old (IQR: 49–68), with the majority of patients being male (58%). The median tumor size was 38 cm (IQR: 28–47).

Patients with WD-GRPLs tended to be younger (median age: 57 (IQR: 43–66) vs. 60 (IQR: 50–71) years old) and more frequently female (43% vs. 41%) compared to those with other histology; however, such differences were not statistically significant (all *p* > 0.05). Conversely, these patients had a similar tumor size (38 (IQR: 28–50) vs. 38 (IQR: 27–43), *p* = 0.6). Descriptive data stratified by histology are summarized in Table 1. Different histologies accounting for “Other Histology” are specified in Supplementary Materials, Table S2.

When stratified by sex category, female patients emerged as being diagnosed with GRPLs at younger ages compared to their male counterparts (56 (IQR: 47–63) vs. 60 (IQR: 52–73), *p* = 0.01)—see Table 2. No additional significant differences were found within our study cohort when stratified by sex category.

### 3.2. Survival Analysis, Cox Proportional Hazards Models and Interaction Analysis

**Figure 1:**
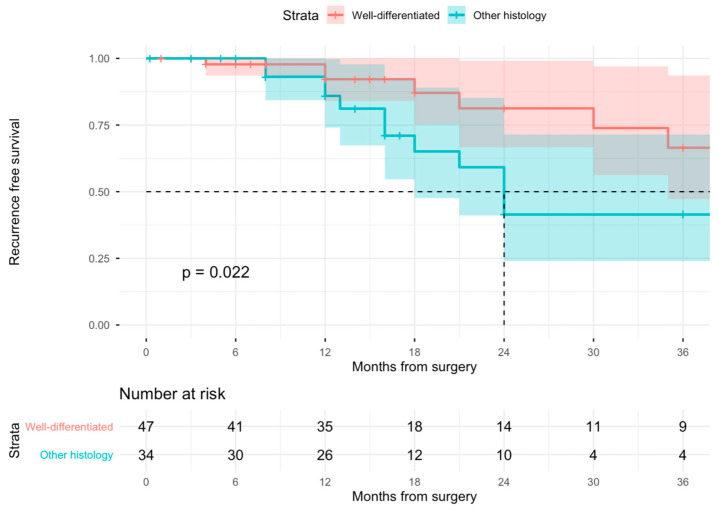
RFS stratified by tumor histology. The dashed line indicates the median survival time.

Within a median (IQR) follow-up of 16 months, a total of 24 patients experienced a recurrence. Kaplan–Meier curves are shown in Figure 1. RFS curves were significantly different between WD and other histology GRPLs (*p* for log-rank test = 0.02). Specifically, at 24 months, RFS was 81% (95% CI: 66–99) in patients diagnosed with a WD-GRPL vs. 41% (95% CI: 24–71%]) in patients diagnosed with other histology.

A Cox multivariable regression model adjusted for tumor dimensions showed that patients diagnosed with other histology had a 3.2-fold increased recurrence risk than their WD counterparts (HR 3.24, 95% CI 1.28–8.17, *p* = 0.03)—see Table 3. Conversely, tumor dimension did not emerge as an independent predictor (HR 1.00, 95% CI 0.85–1.18, *p* > 0.9). In our exploratory interaction analysis, no differential effect was observed in patients between histological subtypes and tumor dimensions (HR 0.90 [0.65–1.25]; *p* = 0.5; Supplementary Materials, Table S3).

## 4. Discussion

GRPLs are mesenchymal tumors arising from the retroperitoneal fatty tissues, able to reach notable dimensions prior to being diagnosed. Surgical resection represents the mainstay of treatment, with tumor size often posing significant technical challenges. However, despite complete resection, recurrence rates remain non-negligible [22,24,25,26]. In this context, histological subtype is a well-established predictor of recurrence, whereas the independent role of tumor size remains controversial. Notably, the impact of size among already giant tumors, along with its interaction with histology, has never been adequately explored. This study aims to address this gap using a large, wide retrospective literature-based cohort.

Our findings were noteworthy. First, female patients received a diagnosis of GRPLs at a younger age compared with male patients; however, this difference was not found to have any impact on recurrence risk (*p* value = 0.11). This finding is particularly interesting when compared to the current literature on liposarcomas epidemiology. According to Bock et al. [5], who analyzed more than 11,162 liposarcoma cases from The Surveillance, Epidemiology, and End Result (SEER) database and 37,499 cases from the National Program of Cancer Registries (NPCR), liposarcomas are tumors which have their peak of incidences between the eighth and ninth decade of life with no differences among sex. Moreover, the specific literature on RPLs [1] highlights a peak of incidence between the sixth and seventh decade of life, with no impact of sex on age at presentation. In this context, our findings may be explained by several factors. Given the relatively indolent behavior of these tumors—particularly those with well-differentiated histology—they may remain undetected for a prolonged period [27,28]. As previously reported in the literature [29], male patients may be less likely to seek medical attention until the onset of clinically relevant symptoms. Similarly, for an equivalent tumor size, the constitutionally higher BMI [30] of male patients may delay the recognition of abdominal swelling compared with their female counterparts.

Second, our analysis showed important differences in recurrence rate between WD and other histology GRPLs without any differential impact of the size of the tumor. Specifically, the 2-year RFS for other histology GRPLs patients in our cohort was 41%, with these patients having a 3.2-fold higher risk of recurrence compared to their WD-GRPLs counterparts. In the multivariable analysis, size did not emerge as a significant predictor of recurrence risk, nor did it have any differential effect depending on the tumor histology. These findings are interesting considering the existing literature. In particular, in their report on 3-year abdominal recurrence of RPLs, Bonvalot et al. [22] assessed a 1.27-fold higher risk of recurrence for other histology when compared to WD RPLs. Despite, not being as impactful as in our cohort, this study points out histology as main predictor of recurrence, as well as tumor grade, margin status and center experience. Of note, tumor size was not a predictor of recurrence in this report either. In this context, our findings suggest a much stronger impact of histology on the recurrence risk of GRPLs compared to normal-size RPLs.

These findings are further contextualized by recent improvements in the characterization of different liposarcoma subtypes. Specifically, Vanni et al. [31] highlighted different biological features which characterized WD liposarcomas. Indeed, WD liposarcomas are characterized by lower CDK4 and MDM2 expression and related alterations compared to their dedifferentiated counterparts, thus directly impacting their prognostic impact. This molecular divergence may at least in part underlie the markedly different recurrence patterns observed in our cohort, reinforcing the notion that histological subtype reflects fundamentally distinct tumor biology rather than merely a categorical distinction.

Despite the intriguing findings reported, our study is not devoid of limitations. First, the database is exclusively built from published case reports and case series, introducing a substantial publication and selection bias. Complex, rare, or surgically challenging cases are inherently more likely to be reported than uneventful clinical presentations, potentially skewing our cohort towards more aggressive disease and higher recurrence rates. Moreover, the majority of the included cases derive from single case reports, further amplifying the impact of publication bias and limiting the representativeness of the cohort. Additionally, selection bias may arise from the fact that only patients with available follow-up data were included in the analysis, which may systematically favor cases with longer or more eventful clinical courses. Taken together, these biases may limit the generalizability of our findings to the broader population of patients with giant retroperitoneal liposarcomas, and our results should therefore be interpreted as hypothesis-generating rather than practice-changing. Prospective multicenter registries specifically designed for rare retroperitoneal tumors will be essential to overcome these limitations and provide more representative evidence. Second, follow-up data are inherently limited by the time of case report publication. To minimize this issue, only patients with available follow-up data were included; however, as noted above, this choice may introduce a degree of selection bias, and results should be interpreted accordingly. Third, our database could not capture the surgical center experience, thus limiting the adjustment of the survival analysis. Fourth, due to the nature of the dataset, our analysis could not correct for other factors possibly driving the higher recurrence rate, namely tumor volume, tumor weight, MDM2/CDK4 and tumor histological heterogeneity. Fifth, the “Other histology” category encompasses distinct liposarcoma subtypes—including dedifferentiated, myxoid, and pleomorphic variants—which differ substantially in terms of biological behavior, aggressiveness, and recurrence potential. Grouping these entities into a single category may therefore obscure relevant differences in outcomes across subtypes, and the observed recurrence risk associated with non-well-differentiated histology should be interpreted with this heterogeneity in mind. For completeness, a recurrence free survival curve of the overall cohort and another stratified by each individual histological subtype have been added in Supplementary Materials, Figures S2 and S3.

## 5. Conclusions

GRPLs are rare tumors in which histology is the most important factor affecting recurrence risk. Conversely, given the already considerable size of these tumors once diagnosed, incremental dimensions seem to have no impact, neither overall nor within specific histological subtypes.

## Figures and Tables

**Table 1:** Descriptive data stratified by histology.

	Overall *N* = 81	Other Histology *N* = 34	Well-Differentiated *N* = 47	*p* ^2^
**Age (years)** ^1^	58 (49–68)	60 (50–71)	57 (43–66)	0.2
**Sex**				>0.9
F	34 (42%)	14 (41%)	20 (43%)	
M	47 (58%)	20 (59%)	27 (57%)	
**Dimension (cm)** ^1^	38 (28–47)	38 (27–43)	38 (28–50)	0.6
**Local Recurrence**	24 (30%)	14 (41%)	10 (21%)	NA

^1^ Median (IQR: Q1–Q3); *N* (%). ^2^ Wilcoxon rank sum test; Pearson’s Chi-squared test.

**Table 2:** Descriptive data stratified by sex category.

	Overall *N* = 81	F *N* = 34	M *N* = 47	*p* ^2^
**Histology**				>0.9
Other Histology	34 (42%)	14 (41%)	20 (43%)	
Well-Differentiated	47 (58%)	20 (59%)	27 (57%)	
**Age (years)** ^1^	58 (IQR: 49–68)	56 (IQR: 47–63)	60 (IQR: 52–73)	0.019
**Dimension (cm)** ^1^	38 (IQR: 28–47)	36 (IQR: 26–45)	40 (IQR: 30–48)	0.3
**Local Recurrence**	24 (30%)	11 (32%)	13 (28%)	0.8

^1^ Median (IQR: Q1–Q3). ^2^ Pearson’s Chi-squared test; Wilcoxon rank sum test.

**Table 3:** The 24-month Cox multivariable regression model adjusted for tumor dimension.

Characteristic	HR	95% CI	*p*
**Histology (other vs. WD)**			
Well-differentiated	—	—	
Other histology	3.24	1.28, 8.17	0.013
**Dimension (×5 cm increase)**	1.00	0.85, 1.18	>0.9
**Sex**			
F	—	—	
M	0.47	0.19, 1.15	0.10

## Data Availability

Data available upon request to santangelo.domenico@hsr.it.

## Supplementary Materials

The following supporting information can be downloaded at: https://www.mdpi.com/article/10.3390/cancers18101649/s1, Figure S1: Inclusion/Exclusion Criteria; Figure S2: Recurrence free survival of the overall cohort; Figure S3: Recurrence free survival stratified per each histological group; Table S1: Comparison of included vs. excluded patients; Table S2: Subcategorization of Other Histology group; Table S3: Interaction analysis between Histology and Dimension.

## Abbreviations

The following abbreviations are used in this manuscript:

GRPLs	Giant retroperitoneal liposarcomas
WD	Well-differentiated
RFS	Recurrence-free survival
