# Supplementary material for: The Impact of Histology Subtype and Size of Giant Retroperitoneal Liposarcomas on Their Risk of Recurrence: A Retrospective Cohort Analysis
Source: Cancers (Basel). 2026 May 20;18(10):1649. doi: 10.3390/cancers18101649 (PMC13204027; doi:10.3390/cancers18101649)
Supplement: Supplementary file 1 [file cancers-18-01649-s001.zip › cancers-4296296-supplementary.pdf]

Supplementary Materials:

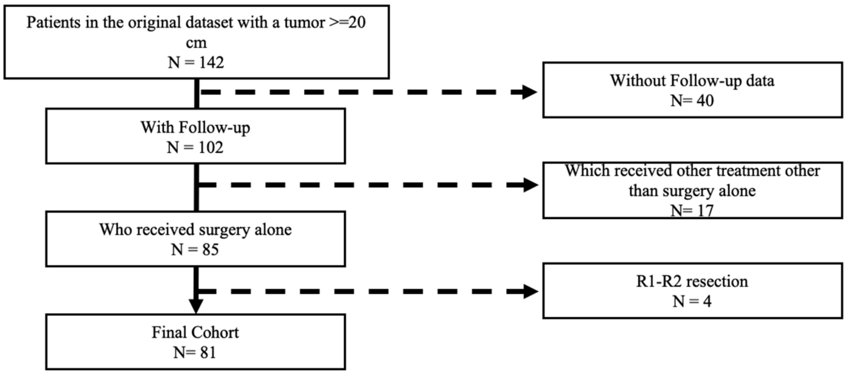

Supplementary Figure S1. Inclusion/Exclusion Criteria.

Supplementary Table S1. **Comparison of included vs. excluded patients.**

Comparison of included vs. excluded patients			
**Characteristic**	**Overall**<br>N = 142[1]	**Excluded**<br>N = 61[1]	**Included**<br>N = 81[1]
Age (years)	58 (IQR: 46–67)	59 (IQR: 45–67)	58 (IQR: 49–68)
Unknown	11	11	0
Sex			
F	61 (43%)	27 (45%)	34 (42%)
M	80 (57%)	33 (55%)	47 (58%)
Unknown	1	1	0
Tumor size (cm)	35 (IQR: 28–45)	33 (IQR: 28–41)	38 (IQR: 28–47)
Histology			
Well differentiated	69 (49%)	22 (36%)	47 (58%)
De-Differentitated	44 (31%)	26 (43%)	18 (22%)
Mixed	14 (9.9%)	3 (4.9%)	11 (14%)
Myxoid	7 (4.9%)	4 (6.6%)	3 (3.7%)
Pleomorph	1 (0.7%)	1 (1.6%)	0 (0%)
Unspecified	7 (4.9%)	5 (8.2%)	2 (2.5%)

[1] Median (IQR: Q1–Q3); n (%)

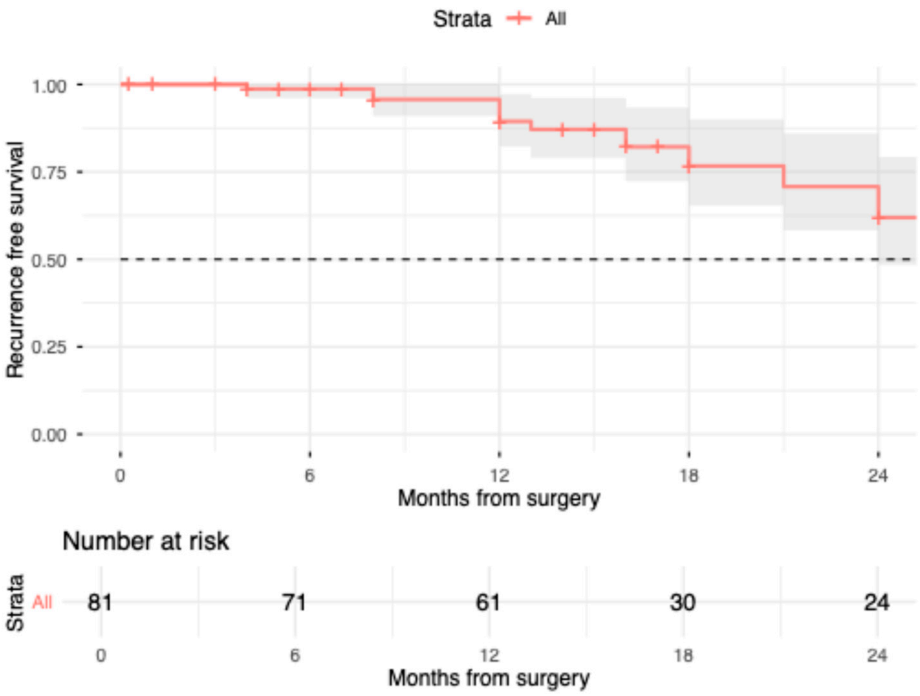

Supplementary Figure S2. Recurrence free survival of the overall cohort.

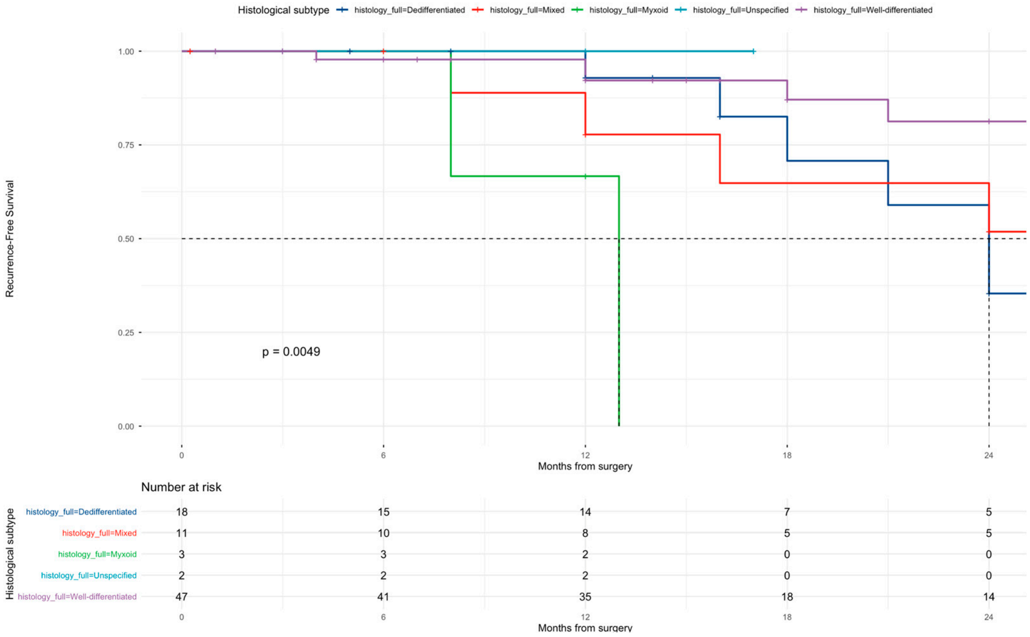

Supplementary Figure S3. Recurrence free survival stratified per each histological group.

Supplementary Table S2. Subcategorization of Other Histology group.

**Other Histology**	**n (%)**
Dedifferentiated	18 (52.9%)
Mixed	11 (32.4%)
Myxoid	3 (8.8%)
Unspecified	2 (5.9%)

Supplementary Table S3. Interaction analysis between Histology and Dimension.

Characteristic	HR	95% CI	p
**Histology**			
Well-differentiated	—	—	
Other histology	7.02	0.50, 98.2	0.15
**Dimension (x 5cm increase)**	1.05	0.84, 1.31	0.7
**Sex**			
F	—	—	
M	0.48	0.20, 1.19	0.11
**Histology x Dimension (x 5cm increase)**			
Other Histology x Dimension (x 5cm increase)	0.90	0.65, 1.25	0.5

Abbreviations: CI = Confidence Interval, HR = Hazard Ratio
